# Identification of the prognostic value of ferroptosis-related gene signature in breast cancer patients

**DOI:** 10.1186/s12885-021-08341-2

**Published:** 2021-05-31

**Authors:** Ding Wang, Guodong Wei, Ju Ma, Shuai Cheng, Longyuan Jia, Xinyue Song, Ming Zhang, Mingyi Ju, Lin Wang, Lin Zhao, Shijie Xin

**Affiliations:** 1 Department of Vascular Surgery, The First Hospital of China Medical University, Shenyang, Liaoning Province China; 2 Department of Pharmacology, School of Pharmacy, China Medical University, Shenyang, Liaoning Province China

**Keywords:** Breast cancer, Ferroptosis, Prognostic signature, Immune status

## Abstract

**Background:**

Breast cancer (BRCA) is a malignant tumor with high morbidity and mortality, which is a threat to women’s health worldwide. Ferroptosis is closely related to the occurrence and development of breast cancer. Here, we aimed to establish a ferroptosis-related prognostic gene signature for predicting patients’ survival.

**Methods:**

Gene expression profile and corresponding clinical information of patients from The Cancer Genome Atlas (TCGA) database and Gene Expression Omnibus (GEO) database. The Least absolute shrinkage and selection operator (LASSO)-penalized Cox regression analysis model was utilized to construct a multigene signature. The Kaplan-Meier (K-M) and Receiver Operating Characteristic (ROC) curves were plotted to validate the predictive effect of the prognostic signature. Gene Ontology (GO) and Kyoto Encyclopedia of Genes, Genomes (KEGG) pathway and single-sample gene set enrichment analysis (ssGSEA) were performed for patients between the high-risk and low-risk groups divided by the median value of risk score.

**Results:**

We constructed a prognostic signature consisted of nine ferroptosis-related genes (ALOX15, CISD1, CS, GCLC, GPX4, SLC7A11, EMC2, G6PD and ACSF2). The Kaplan-Meier curves validated the fine predictive accuracy of the prognostic signature (*p* < 0.001). The area under the curve (AUC) of the ROC curves manifested that the ferroptosis-related signature had moderate predictive power. GO and KEGG functional analysis revealed that immune-related responses were largely enriched, and immune cells, including activated dendritic cells (aDCs), dendritic cells (DCs), T-helper 1 (Th1), were higher in high-risk groups (*p* < 0.001). Oppositely, type I IFN response and type II IFN response were lower in high-risk groups (*p* < 0.001).

**Conclusion:**

Our study indicated that the ferroptosis-related prognostic signature gene could serve as a novel biomarker for predicting breast cancer patients’ prognosis. Furthermore, we found that immunotherapy might play a vital role in therapeutic schedule based on the level and difference of immune-related cells and pathways in different risk groups for breast cancer patients.

**Supplementary Information:**

The online version contains supplementary material available at 10.1186/s12885-021-08341-2.

## Background

Breast cancer (BRCA) is the most common cancer among women worldwide, with high morbidity and mortality rates [1]. According to breast cancer statistics, there are expected to be about 276,480 new cases of invasive breast cancer and 42,170 deaths for women in the United States in 2020 [2]. Despite major advances in therapies, including surgery, radiation therapy, chemotherapy, hormonal therapy, targeted and immunotherapy, the mortality rate for breast cancer still remains high [3]. It has been reported that the immune microenvironment was crucial to the development of breast cancer, especially the infiltration of immune cells. These immune cells either expressed different immune antigens for themselves or influenced other immune cells to help tumor cells escape immunity, but specific mechanism still isn’t very clear [4]. Therefore, to determine the molecular mechanism of breast cancer occurrence and development is crucial to advance cancer therapies.

Ferroptosis is a novel form of regulated cell death characterized by destruction of intracellular redox balance and non-apoptosis [5]. Ferroptosis has become a promising therapeutic option for triggering cancer cell death [6]. It is reported that siramesine and lapatinib are effective ferroptosis inducers in breast cancer [7, 8]. In addition, Dihydroisonone I (DT), a pure compound present in Salvia miltiorrhiza, can inhibit GPX4 protein expression and induce ferroptosis through lipid peroxidation to improve the prognosis of breast cancer [9]. In terms of mechanism, elastin induces ferroptosis in breast cancer via the Glycogen synthase kinase-3β (GSK3β) / (nuclear factor erythroid 2-related factor 2) Nrf2 signaling pathway [10]. Besides, acyl-CoA synthetase long-chain family member 4 (ACSL4) can increase the content of intracellular lipids to promote ferroptosis [11]. Another study shows that the down-regulation of activator of transcription factor 2 (ATF2) promote ferroptosis by a negative feedback manner [12]. However, whether these ferroptosis-related genes are associated with the prognosis of BRCA patients remains non-statistical. And immunological therapy is also not ignored for breast cancer patients.

Immune checkpoint blockade therapy has been used for a variety of cancers, including breast cancer, most of which are specific to CTLA-4 and PD-1/ PD-L1 [13]. And related drugs have been developed for clinical application, including Tremelimumab, Ipilimumab, Avelumab, Atezolizumab and Pembrolizumab. Meanwhile, now a number of new immune checkpoint inhibitors have emerged, such as LAG-3, TIM-3 and TIGIT. And targeted drugs were also in clinical trials [14]. However, sometimes a single treatment never met our expectations for the desired effect. Thus, we have developed a combination of therapies to combat the onset and progression of tumors, such as chemotherapy and immunotherapy or radiotherapy and immune checkpoint blocking therapy [14].

In this study, we first acquired mRNA expression data and corresponding clinical profiles of BRCA patients from The Cancer Genome Atlas (TCGA). Then, we constructed a prognostic multigene signature with ferroptosis-related differentially expressed genes (DEGs), and validated the predictive power of prognostic signature. Meanwhile, we performed GEO database to verify the credibility. Finally, we further conducted functional enrichment analysis to explore the potential immune-related mechanisms.

## Methods

### Data collection

Gene expression data (count) and corresponding clinical information of 1223 breast cancer patients were obtained from The Cancer Genome Atlas (TCGA) up to September 08, 2020 (https://portal.gdc.cancer.gov/repository). This cohort has 1097 breast cancer patients with the associated gene expression profiles and clinical characteristics. Then, 21 patients were removed due to transcriptomic and clinical data was incomplete. Thus, the remaining data (*n* = 1076) with complete follow-up information was included in our training data set for further analyses. The testing data set for validation was downloaded from the Gene Expression Omnibus database (GEO, https://www.ncbi.nlm.nih.gov/geo/). GSE42568 was conducted by GPL570 (Affymetrix Human Genome U133 Plus 2.0 Array), including 104 tumor samples with breast cancer and 17 non-tumor samples as a normal control. The ICGC database (https://icgc.org/) was also downloaded to verify the reliability of the model. Furthermore, 60 ferroptosis-related genes were retrieved from the previous literature and are provided in Supplementary Table S1 [5].

### Construction and validation of a prognostic ferroptosis-related gene signature

The “limma” R package was performed to ascertain DEGs related to ferroptosis between tumor tissues and non-tumor tissues with a false discovery rate (FDR) < 0.05 in the TCGA cohort. Univariate Cox analysis of overall survival (OS) was used to screen ferroptosis-related genes with prognostic values and was visualized by Forest plots. The intersection of ferroptosis-related DEGs and prognostic genes was demonstrated by Venn diagram and was visualized by heatmap. An interaction network of prognostic DEGs was generated by Search Tool for the Retrieval of Interacting Genes (STRING) database (https://string-db.org). We performed Human Protein Atlas (HPA) database (http://www.proteinatlas.org/) to evaluated the expression of DEGs. The Least absolute shrinkage and selection operator (LASSO)-penalized Cox regression analysis was used to construct a prognostic model for minimizing the risk of overfitting by performing the function “glmnet” of R package. Subsequently, the risk score of patients was calculated based on gene expression and corresponding Cox regression coefficient as follows: score = e^sum (each gene’s expression × corresponding coefficient)^ (Table S2). Then, patients were divided into high-risk and low-risk groups according to the median risk score values. Based on the expression of genes signature, PCA was performed with the “prcomp” function of the “stats” R package. Furthermore, t-SNE were applied to explore the distribution of different groups using the “Rtsne” R package. Survival analysis between high-risk and low-risk groups was carried out by the “surv_cutpoint” function of the “survminer” R package. Time-dependent ROC curve analyze was performed by “survivalROC” R package to evaluate the predictive accuracy of the genes signature.

### Functional enrichment analysis

Gene Ontology (GO) and Kyoto Encyclopedia of Genes and Genomes (KEGG) pathway enrichment analysis were performed for patients between the high-risk and low-risk groups by using the “clusterProfiler” R package. GO terms and KEGG pathways with *P* values < 0.05 were statistically significant. The infiltrating score of 16 immune cells and the activity of 13 immune-related pathways were determined by the “single-sample gene set enrichment analysis (ssGSEA) “ function of the “gsva” R package (Table S3).

### Statistical analysis

Statistical analyses were performed by using R (version 4.0.2) software packages. Perl language was used for data matrix and all data processing. A two-tailed *P* < 0.05 was considered statistically significant.

## Results

**Fig. 1:**
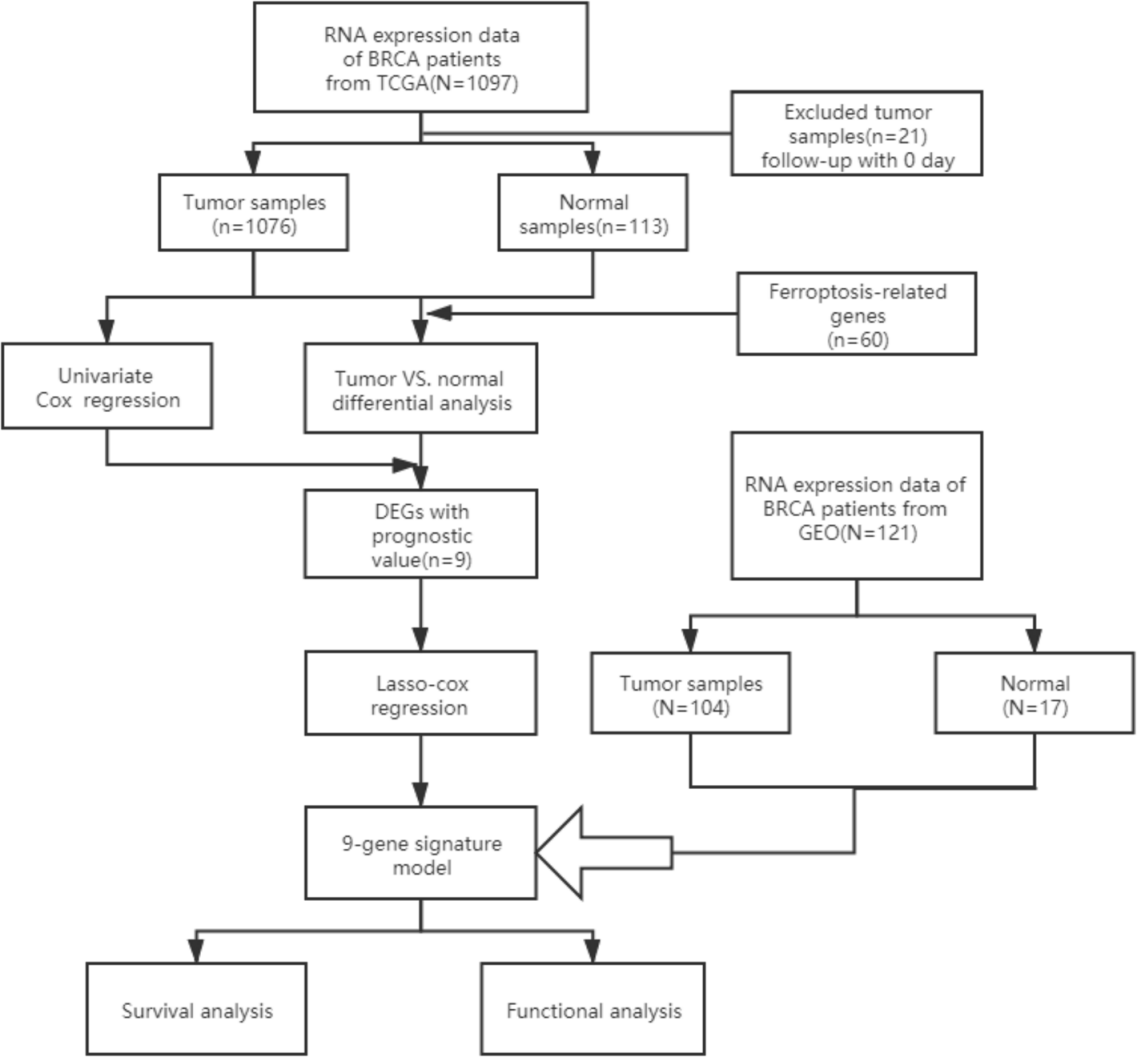
Overview of the process of this study

The detailed flowchart is shown in Fig. 1. In this study, a total of 1076 BRCA patients from the TCGA cohort were finally enrolled. The general clinical information of those patients was provided in Table 1.

**Table 1:** Clinical pathological parameters of patients with breast cancer in this study

	TCGA cohort
**No. of patients**	1076
**Age (median)**	58
**Sex (%)**	
Female	1076 (100%)
Male	0 (0%)
**Stage/grade (%)**	
I/1	183 (17%)
II/2	608 (56.5%)
III/3	242 (22.5%)
IV	20 (1.9%)
Unknown	23 (2.1%)
**ER status (%)**	
Positive	806
Negative	240
Unknown	30
**PR status (%)**	
Positive	698
Negative	345
Unknown	33
**Her2 status (%)**	
Positive	161
Negative	564
Unknown	351
**Therapy**	
Chemotherapy	490
Immunotherapy	4
Hormone Therapy	269
Targeted Molecular therapy	5
Other	308
**Survival status OS days (median)**	1256

### Identification of prognostic ferroptosis-related DEGs in TCGA

**Fig. 2:**
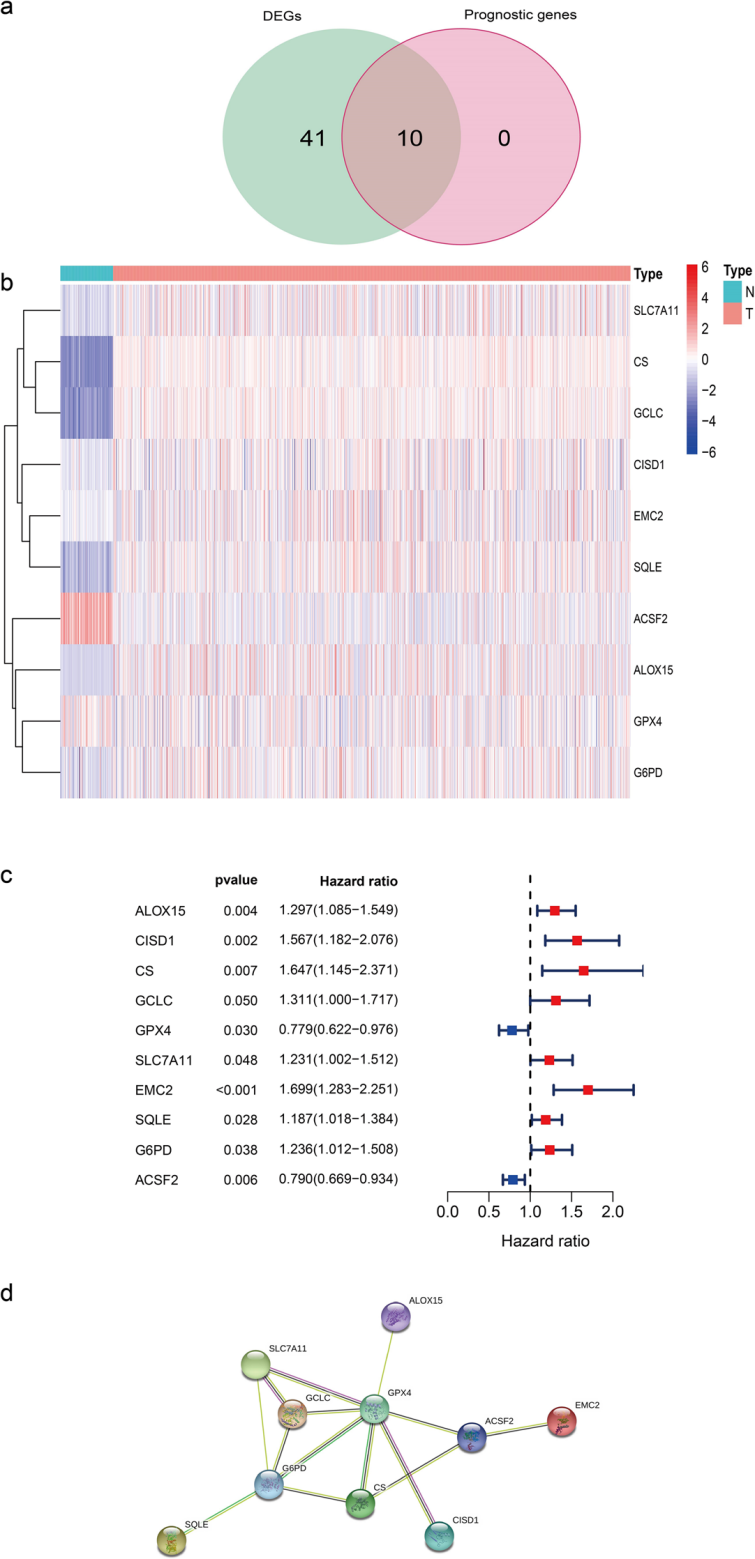
Identification of the candidate ferroptosis-related genes in TCGA. **a** Venn diagram showed ferroptosis-related differentially expressed genes between tumor and adjacent normal tissue that were correlated with OS. **b** Tumor tissue contained eight upregulated genes and two downregulated genes. **c** Forest plots to show the results of the univariate Cox regression analysis between gene expression and OS. **d** The PPI network revealed the interactions among the candidate genes and excavate the hub genes

Most of the ferroptosis-related genes (51/60, 85%) were differentially expressed between tumor tissues and adjacent nontumorous tissues, and all of them were associated with OS in the univariate Cox regression analysis (Fig. 2a). Upregulated genes, including 15-lipoxygenase (ALOX15), CDGSH iron sulfur domain 1 (CISD1), citrate synthase (CS), glutamate-cysteine ligase catalytic subunit (GCLC), cystine/glutamate antiporter solute carrier family 7 member11 (SLC7A11), estrogen receptor membrane complex 2 (EMC2), squalene epoxidase (SQLE) and glucose-6-phosphate dehydrogenase (G6PD), manifested an excellent prognosis in heatmap (Fig. 2b) and univariate Cox analysis (Fig. 2c). The protein-protein interaction network among these genes illuminates that GPX4 and G6PD were the hub gene (Fig. 2d).

### The expression of six-gene is higher in BRCA tissue compared with normal tissue in HPA database

**Fig. 3:**
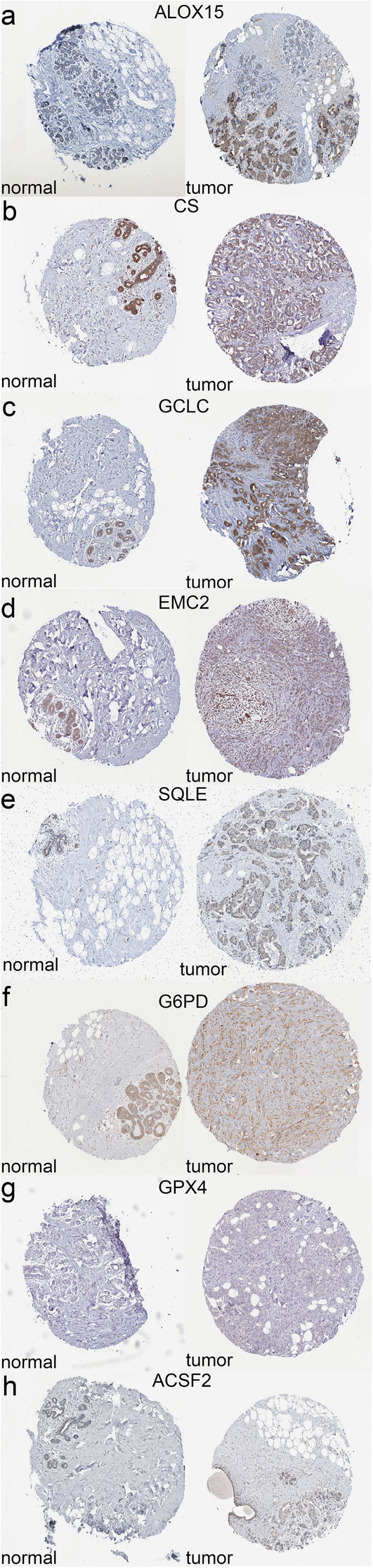
The expression of candidate signatures in both BRCA tissue and normal tissue in HPA database. **a**-**f** The expression of six genes (ALOX15, CS, GCLC, EMC2, SQLE, G6PD) is higher in tumor tissue. **g** and **h** The expression of two genes (GPX4, ACSF2) isn’t significant difference

According to the immunohistochemical analyses in HPA database, the high staining intensity of these six genes (ALOX15, CS, GCLC, EMC2, SQLE, G6PD) in BRCA tissues contrasted starkly with the low intensity or lack of staining in normal tissues (Fig. 3a-f), while GPX4 and ACSF2 (Fig. 3g-h) didn’t show striking difference. Regrettably, CISD1 and SLC7A11 didn’t be founded in HPA database.

### Construction and evaluation of nine-gene prognostic model in TCGA

**Fig. 4:**
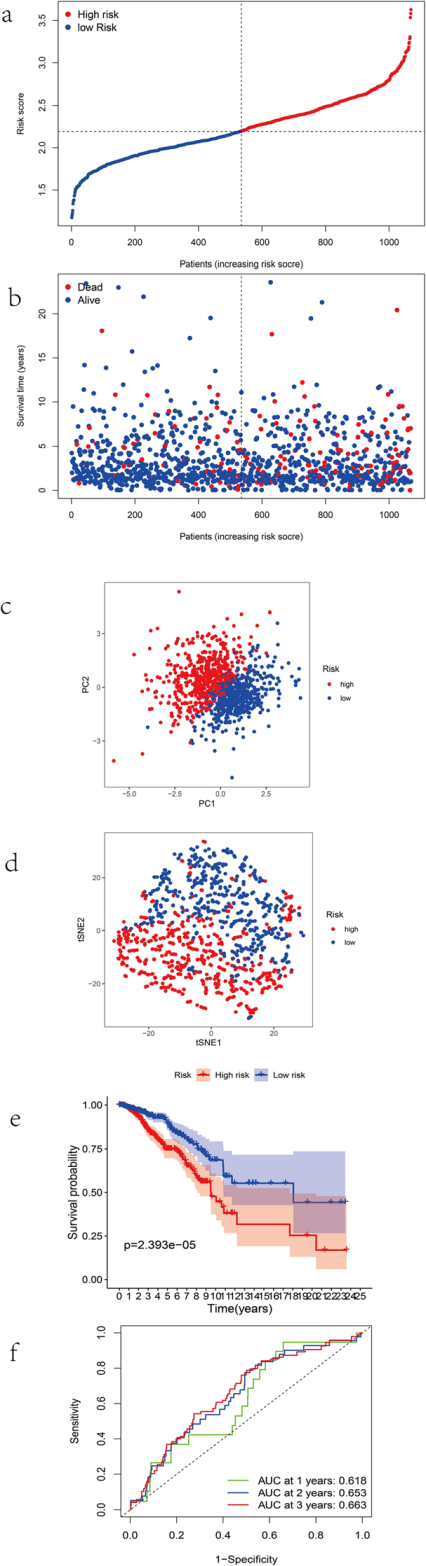
Prognostic analysis of the 9-gene signature model in TCGA. **a** The distribution and median value of the risk scores. **b** The distributions of OS status, OS and risk scores. **c** and **d** PCA and t-SNE analysis plot. **e** Kaplan-Meier survival curves of OS of high-risk group and low-risk group. **f** AUC of time-dependent ROC curves verified the predictive power of the risk score

The LASSO Cox regression analysis was applied to establish a prognostic model using the expression profile of the 10 genes mentioned above. Finally, a nine-gene (15-lipoxygenase (ALOX15), CDGSH iron sulfur domain 1 (CISD1), citrate synthase (CS), glutamate-cysteine ligase catalytic subunit (GCLC), selenoenzyme glutathione peroxidase (GPX4), cystine/glutamate antiporter solute carrier family 7 member11 (SLC7A11), estrogen receptor membrane complex 2 (EMC2), glucose-6-phosphate dehydrogenase (G6PD) and acyl-CoA synthetase family member2 (ACSF2)) prognostic model was constructed to predict prognosis based on the risk score = e (0.167 * expression level of ALOX15 + 0.212 * expression level of CISD1 + 0.228 * expression level of Cs + 0.057 * expression level of GCLC + (− 0.116) * expression level of GPX4 + 0.018 * expression level of SLC7A11 + 0.211 * expression level of EMC2 + 0.134 * expression level of G6PD + (− 0.098) * expression level of ACSF2). The patients were divided into a high-risk group (*n* = 535) or a low-risk group (*n* = 535) according to the median value of risk score in TCGA cohort (Fig. 4a), Principal component analysis (PCA) and t-distributed stochastic neighbor embedding (t-SNE) analysis suggested the patients in different risk groups were distributed in two directions (Fig. 4b-c). The patients in high-risk group had a higher probability of death earlier than those in low-risk group (Fig. 4d). Analogously, the Kaplan-Meier curve showed the prognostic signature clearly distinguished patients with high and low survival rate (Fig. 4e). The area under the curve (AUC) confirmed that the identified prognostic signature had a robust efficiency for predicting the OS for BRCA patients (AUC = 0.618, 0.653 and 0.663; at 1, 2 and 3 year, respectively, Fig. 4f).

### Validation of the nine-gene signature in GEO and ICGC dataset

**Fig. 5:**
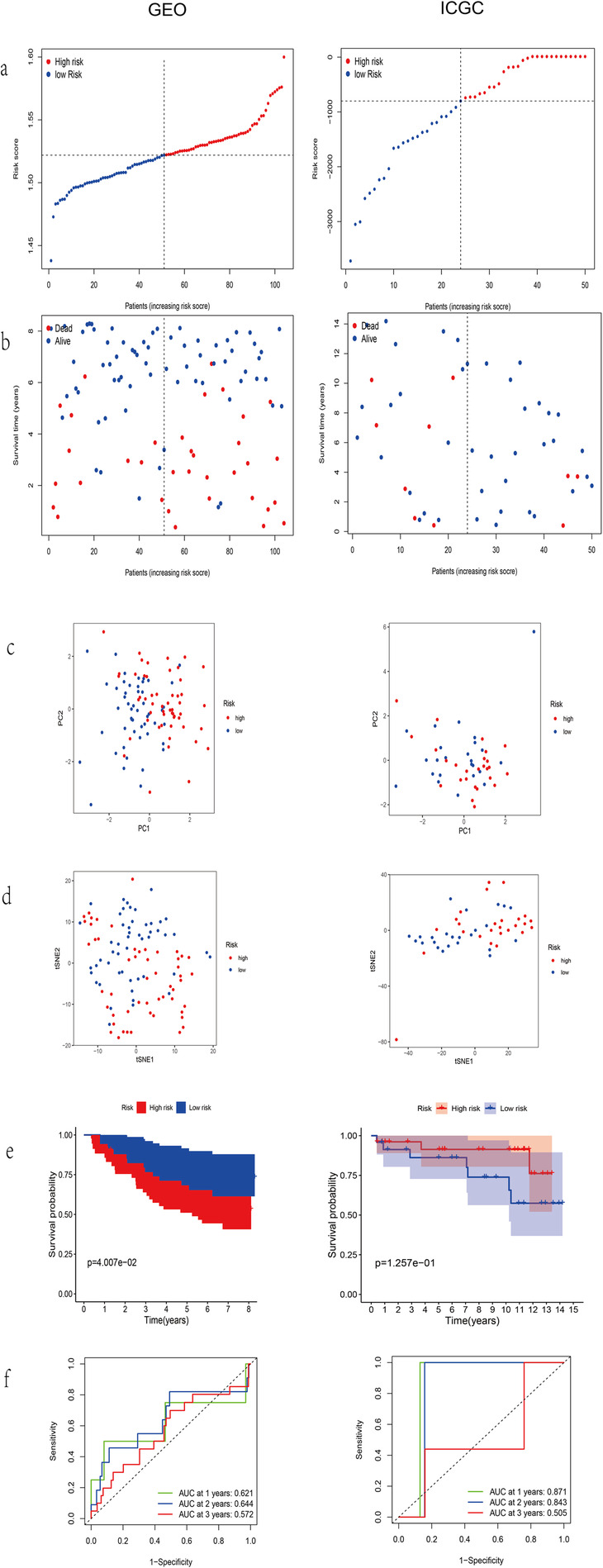
Validation of the 9-gene signature model in GEO and ICGC. **a** The distribution and median value of the risk scores. **b** The distributions of OS status, OS and risk scores. **c** and **d** PCA and t-SNE analysis plot. **e** Kaplan-Meier survival curves of OS of high-risk group and low-risk group. **f** AUC of time-dependent ROC curves verified the predictive power of the risk score

The breast cancer patients from the GEO and ICGC cohort were categorized into high-risk or low-risk groups by the median value of risk score (Fig. 5a). The results of PCA and t-SNE analysis in GEO and ICGC was similar to TCGA (Fig. 5b-c). The high-risk group had a poor prognosis compared to the low-risk group (Fig. 5d). the KM curve showed the prognostic signature clearly distinguished patients with different survival rate (Fig. 5e). The AUC of ROC indicated nine-gene signature had a moderate predictive ability (AUC = 0.621, 0.644 and 0.572; at 1, 2 and 3 year, respectively in GEO. AUC = 0.871, 0.843 and 0.505; at 1, 2 and 3 year, respectively in ICGC, Fig. 5f).

### Independent prognostic value of the nine-gene signature in TCGA

**Fig. 6:**
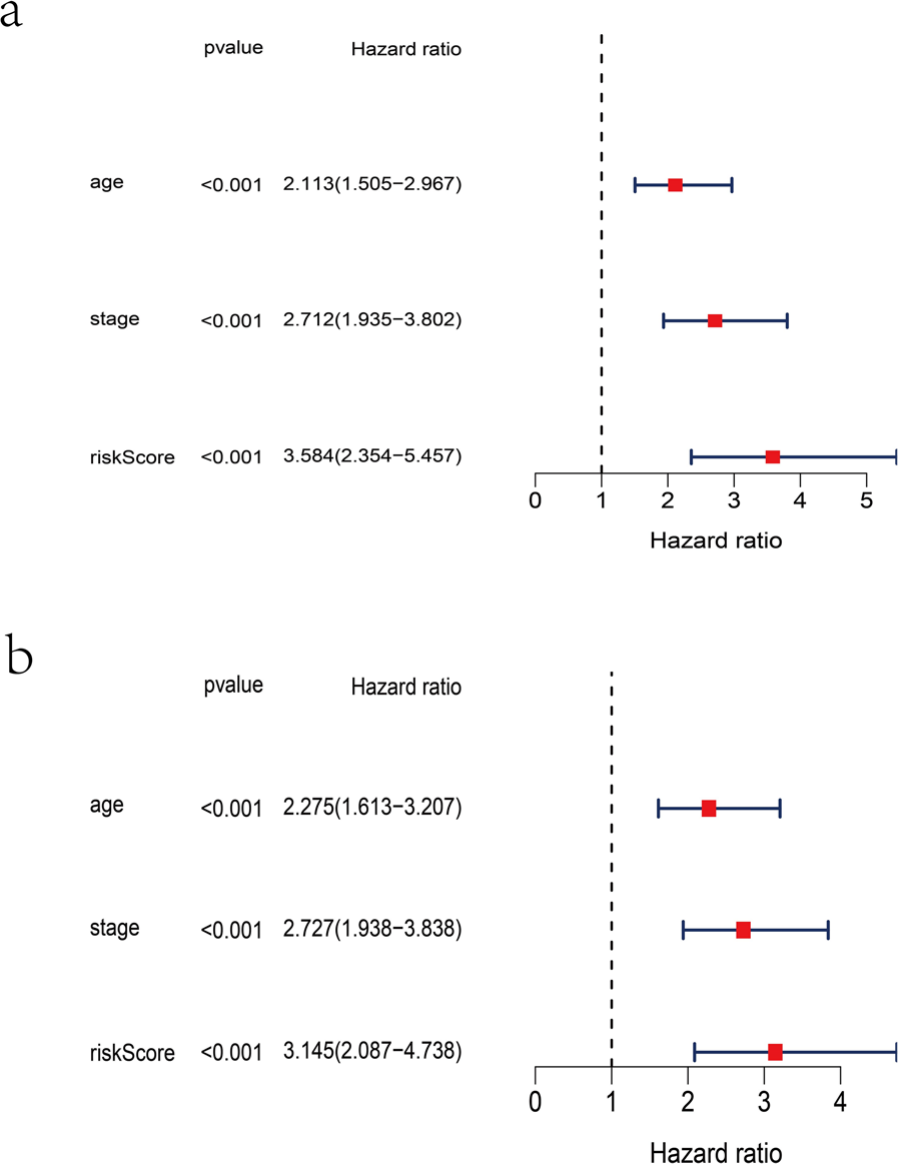
The results of the univariate and multivariate Cox regression analyses regarding significant survival-related clinicopathological parameters in TCGA

In order to investigate the independence of prognostic signature for OS, we performed univariate and multivariate Cox regression tests to determine the relationship between the risk model and different clinicopathological parameters (Fig. 6a-b). The risk score was significantly associated with OS in univariate Cox regression analyses (HR = 3.584, 95% CI = 2.353–5.457, *P* < 0.001). Consistently, the risk score still indicated to be an independent predictor for OS in the multivariate Cox regression analysis (HR = 3.145, 95% CI = 2.087–4.738, *P* < 0.001) (Table 2).

**Table 2:** Univariate and multivariate Cox regression analysis between prognostic risk model and clinicopathological parameters

Characteristics	Univariate Cox regression analysis				Multivariate Cox regression analysis			
	HR	HR.95 L	HR.95H	*p* value	HR	HR.95 L	HR.95H	*p* value
**Age**	2.162465653	1.473275815	3.174054478	< 0.001	1.611064335	0.966106504	2.686586088	0.068
**Stage**	2.30704471	1.563193243	3.404860733	< 0.001	5.97890086	3.483108014	10.26303386	< 0.001
**T**	1.518821112	0.972301454	2.372533293	0.066	1.231451	0.58982	2.510992	0.55
**N**	2.213785821	1.419917467	3.451501778	< 0.001	0.392161053	0.213503048	0.720318953	0.003
**M**	2.993734856	1.097895745	8.163296402	0.032	2.370712367	0.602318224	9.331076006	0.217
**Risk score**	3.58086558	2.215190038	5.788486803	< 0.001	2.61272202	1.365950383	4.997484859	0.004

### Immune-related functions and pathways were enriched in GO and KEGG

**Fig. 7:**
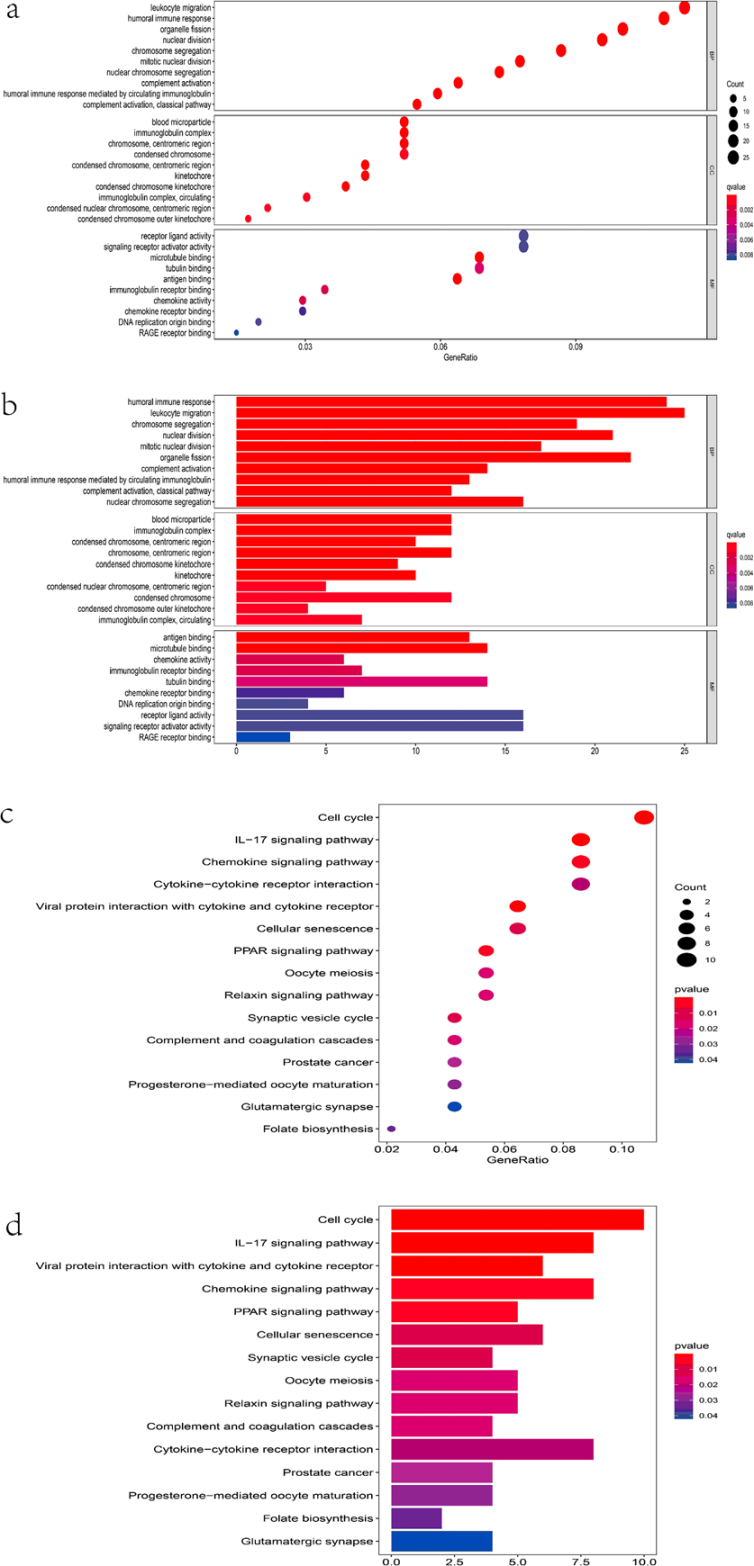
Representative results of GO and KEGG analyses in TCGA. **a** and **b** The results of GO biological process enrichment, GO cellular component enrichment and GO molecular function enrichment of DEGs. **c** and **d** The results of KEGG pathways analysis of DEGs

GO enrichment and KEGG pathway analyses were performed to elucidate the biological functions and pathways that were associated with the risk score. GO analysis results showed that several immune -related molecular functions were significantly enriched (Fig. 7a-b). Likewise, KEGG analyses still found that genes were significantly enriched in IL-17 signaling and cytokine-cytokine receptor interaction pathway (Fig. 7c-d).

### The distribution of immune-associated cells and processes in high- and low-risk group

**Fig. 8:**
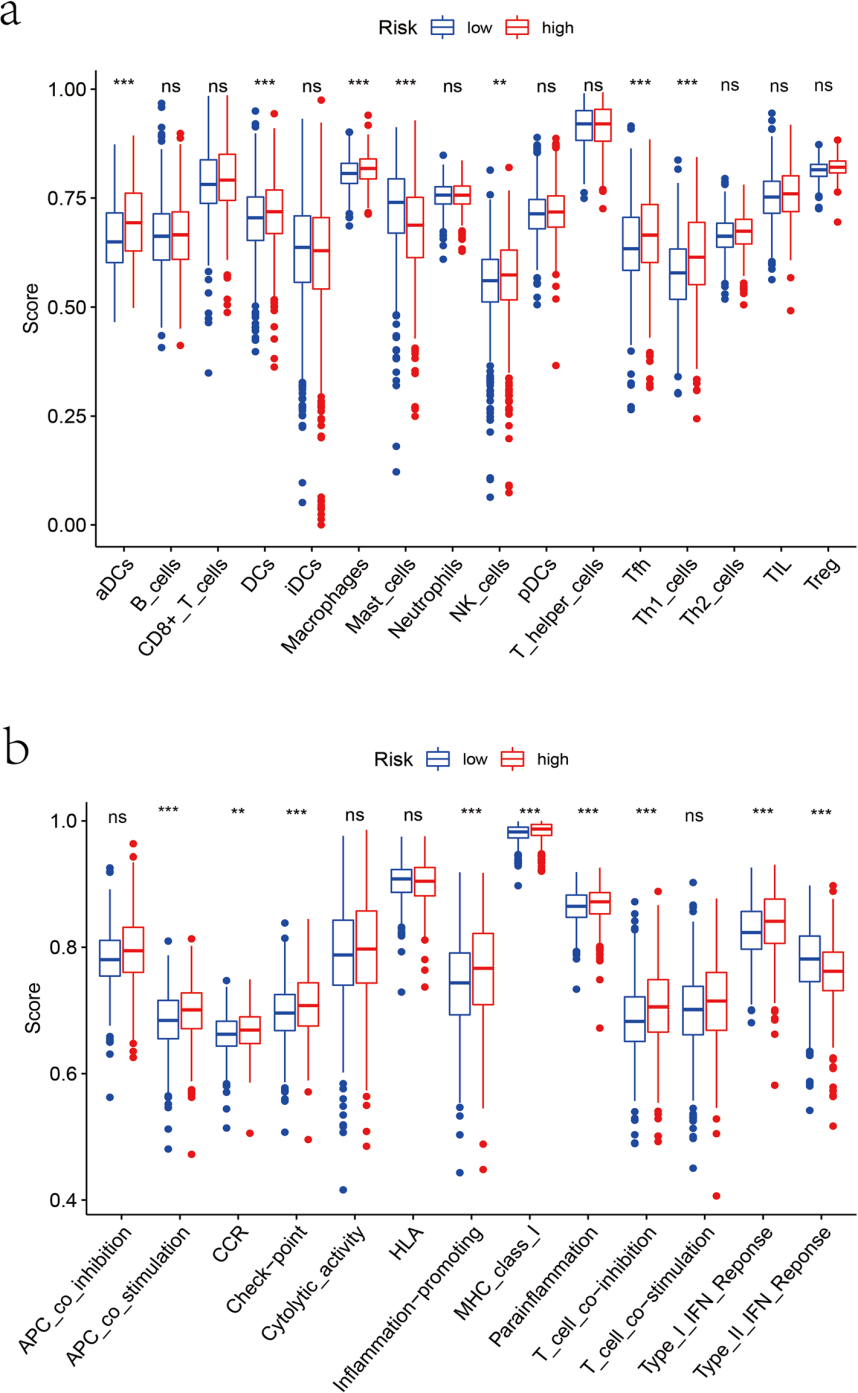
The results of the ssGSEA scores between different risk groups in TCGA. **a** The upper boxplots displayed the scores of 16 immune cells. **b** The under boxplots displayed the scores of 13 immune-related functions. Adjusted *P* values were showed as: ns, not significant; *, *P* < 0.05; **, *P* < 0.01; ***, *P* < 0.001

To further explore the relevancy between the risk score and immune status, we quantified the enrichment scores of diverse immune cell subpopulations, associated functions or pathways with ssGSEA. Contents of the antigen presentation process, including the score of activated dendritic cells (aDCs), dendritic cells (DCs), antigen presenting cell (APC) co-stimulation, inflammation-promoting and MHC class-I (Fig. 7a-b), were significantly different between the high-risk and low-risk group. Moreover, the score of macrophages, natural killer cell (NK), T follicular helper cell (Tfh), T-helper 1 (Th1), T-cell-co − inhibition, type I IFN response, and type II IFN response were higher in the high-risk group, while the scores of mast cells was just the opposite (Fig. 8a-b). It is reported that the occurrence of breast cancer is closely related to the infiltration of immune cells. In the early stage, immune cells played a role in eliminating tumors and gradually were depleted, eventually leading to the weakening of the immune response [15, 16]. Our results might explain the occurrence of early breast cancer, the greater the threat of tumors to human body, the more serious the immune response.

## Discussion

Breast cancer is an extremely common malignancy in women and has a poor prognosis. In recent years, new biomarkers have emerged for the prognosis of cancer patients [17], but this is the first time to discuss the establishment of a prognostic model related to ferroptosis in breast cancer patients.

The prognostic model presented in this study consist of nine ferroptosis-related genes (ALOX15, CISD1, CS, GCLC, GPX4, SLC7A11, EMC2, G6PD and ACSF2). Many studies reported that these genes played an significant role in the pathogenesis of breast cancer. ALOX15 mediates lymphatic vessel invasion and lymph node metastasis in human breast cancer xenograft mouse [18]. CISD1 promotes human breast cancer proliferation and confers autophagy resistance [19, 20]. CS restrains aggressive triple-negative breast cancer cells by targeting glycolysis and the cancer stem cell phenotype [21]. 15-deoxy-Δ (12,14)-prostaglandin J2-induced GCLC is mediated by Multidrug resistance-associated protein 1 via Nrf2 signaling in human breast cancer cells [22]. GPX4, as an oncogene, inhibits the ferroptosis effect of cancer cells, while GPX4 inhibition can enhance the anticancer effect of cisplatin [23]. SLC7A11 over-expression promotes lipo-ROS accumulation in MCF-7 breast cancer cells [24]. The blockade of G6PD by autophagy enhanced the inhibitory effect of tyrosine kinase on breast cancer cells [25]. Although these genes are closely related to the pathogenesis of breast cancer, for the first time we have combined them as a marker of prognosis for BRCA patients.

In this study, we established nine genetic biomarkers as a new prognostic model and analyzed their ability to predict the prognosis of high-risk and low-risk groups. The prognostic performance of the model was verified by KM curve and ROC curve, and the results showed that the model had good predictive performance. Meanwhile, we also drew forest plot with different clinical parameters, and the results showed that there were significant correlations between them. Prognostic indicators were also good independent indicators of survival after adjustment for clinical parameters, including age (≤60 years or >60 years), cancer stage (stage I-II or stage II-IV), risk Score (high or low). The above confirmed that the model can predict the prognostic characteristics of breast cancer patients.

Based on DEGs between different risk groups, we performed GO and KEGG functional analyses, and found unexpectedly that many immune-related biological processes and pathways were enriched [16, 26]. Therefore, we further explored the function of immune cell function between the high-risk and low-risk groups and different subgroups. The results showed that most of immune-related cells and functions possessed a higher risk score. This might be related to the overactivation of the immune system in the early stages of breast cancer, for specific performance that the level of antigen presentation and Th1 cells were higher in high-risk groups, which suggested that relative genes were also significantly altered. Therefore, these targets might provide a possibility for immunotherapy of breast cancer patients.

## Conclusion

In conclusion, this study established a new prognostic model associated with nine ferroptosis-related genes and the good prediction ability of the model was verified by three databases, including TCGA、GEO、ICGC database. Besides, we found immune-related cells and pathways were significant differences in high- and low-risk group, which might be helpful for illustrating the application of immunotherapy for breast cancer patients.

## Supplementary Information

**Additional file 1: Table S1.** 60 ferroptosis-related genes were retrieved from the previous literature.

**Additional file 2: Table S2.** Coefficient values of nine selected genes.

**Additional file 3: Table S3.** Genes of 16 immune cells and 13 immune-related pathways.

## Data Availability

The datasets used and/or analyzed during the current study are available from the corresponding author on reasonable request.

## Abbreviations

BRCA: Breast cancer
DEG: Expressed gene
FDR: False discovery rate
OS: Overall survival
TCGA: The Cancer Genome Atlas
GEO: Gene Expression Omnibus
ICGC: International Cancer Genome Consortium
HPA: Human Protein Atlas
STRING: Search Tool for the Retrieval of Interacting Gene
LASSO: The Least absolute shrinkage and selection operator
K-M: Kaplan-Meier
ROC: Receiver operating characteristic
AUC: Area under the curve
GO: Gene ontology
KEGG: Kyoto Encyclopedia of Genes, Genomes
ssGSEA: Single-sample gene set enrichment analysis
ALOX15: 15-lipoxygenase
CISD1: CDGSH iron sulfur domain 1
CS: Citrate synthase
GCLC: Glutamate-cysteine ligase catalytic subunit
SLC7A11: Solute carrier family 7 member11
EMC2: Estrogen receptor membrane complex 2
SQLE: Squalene epoxidase
G6PD: Glucose-6-phosphate dehydrogenase
APC: Antigen presenting cell
NK: Natural killer cell
aDCs: Activated dendritic cells
DCs: Dendritic cells
Th1: T-helper 1
Tfh: T follicular helper cell
DT: Dihydroisonone I
GSK3β: Glycogen synthase kinase-3β
Nrf2: Nuclear factor erythroid 2-related factor 2
ACSL4: Acyl-CoA synthetase long-chain family member 4
ATF2: Activator of transcription factor 2
CTLA-4: Anti-cytotoxic T lymphocyte-associated antigen-4
PD-1/PD-L1: Anti-programmed cell death protein 1/programmed cell death ligand 1
LAG-3: Lymphocyte activation Gene-3
TIM-3: T cell immunoglobulin and mucin-domain containing-3
TIGIT: T cell immunoglobulin and ITIM domain
